# Measuring anxiety & depression in the post critical care population - comparison of the hospital anxiety and depression scale and intensive care psychological assessment tool

**DOI:** 10.1186/2197-425X-3-S1-A963

**Published:** 2015-10-01

**Authors:** N Mason, D Egan, S O'Loghlen, D Conway

**Affiliations:** Manchester Royal Infirmary, Central Manchester University Hospitals NHS Foundation Trust, Manchester Academic Health Science Centre, Manchester, United Kingdom

## Introduction

The impact of critical illness on psychological wellbeing can be severe with patients reporting high levels of anxiety & depression [1]. The psychological impact of critical illness can be long lasting, affecting both patients and their families and impacting on quality of life following hospital discharge [2]. A minority of patients will develop post-traumatic stress disorder following the acute illness and the NICE guideline CG83 recommends that patients are assessed for acute distress and risk of future psychological morbidity [3].

The Hospital Anxiety and Depression Scale was developed as a snapshot assessment of psychological morbidity for hospital in-patients and has been validated in Critical Care Follow Up Clinic patients with a cut-off of ≥8 /14 for each domain for mild distress and ≥11/14 for significant morbidity [4].

The IPAT tool has been validated in critically ill patients as a screening tool to detect acute distress in critical care patients once they are awake, alert & orientated, with a cut off ≥7 / 20 indicating morbidity [5]. It has not been previously reported immediately following Critical Care discharge.

## Objectives

The aims of this survey are to measure and compare screening of acute distress in patients recovering from critical illness recently transferred to the general ward, using two different assessment tools - HADS and IPAT

## Methods

Prospective data was collected from patients within 48 hours of discharge from the critical care unit in patients who received any level 3 critical care or level 2 critical care for greater than 5 days. The assessment tools were administered by a trained nurse to all patients who were awake and could comprehend the questions. Data were collected between December 2014 - January 2015 and uploaded to an electronic database as part of routine care in accordance with NICE CG83 and analysed with Stats Direct.

## Results

68 patients completed HADS & IPAT simultaneously. 7 patients scored ≥8 & 5 ≥11 for HAD Anxiety. 12 scored ≥8 & 7 ≥11 for HAD depression. 10 scored ≥11 on the IPAT. Correlation of Total HADS and IPAT scores r = 0.710526. The median time taken to administer HADS was 4 minutes and IPAT 2 minutes. 3 patients scored ≥8 HAD anxiety yet did not score ≥7 on IPAT.

## Conclusions

IPAT can be administered to most patients following critical care discharge. There was reasonable correlation with HADS. IPAT scored low in patients with no recollection of ICU even when HAD anxiety score was elevated. This merits further investigation.

